# Correction: The cross-sectional association of parental psychosocial status with children’s Body Mass Index z-score and the mediating role of children’s energy balance behaviors—the ABCD Study

**DOI:** 10.1371/journal.pone.0305784

**Published:** 2024-06-13

**Authors:** Meredith L. Overman, Tanja Vrijkotte, Yolanda M. Sánchez Castro, Margreet W. Harskamp-van Ginkel, Monica Hunsberger, Carry M. Renders, Stef P. J. Kremers, Mai J. M. Chinapaw

There are errors in the Author Contributions. The correct contributions are:

**Conceptualization:** Meredith L. Overman, Tanja Vrijkotte, Yolanda M. Sánchez Castro, Margreet W. Harskamp-van Ginkel, Monica Hunsberger, Mai J.M. Chinapaw

**Data curation:** Tanja Vrijkotte

**Formal analysis:** Meredith L. Overman, Tanja Vrijkotte, Yolanda M. Sánchez Castro, Margreet W. Harskamp-van Ginkel, Mai J.M. Chinapaw

**Funding acquisition:** Tanja Vrijkotte, Carry M. Renders, Stef P.J. Kremers, Mai J.M. Chinapaw

**Investigation:** Tanja Vrijkotte

**Methodology:** Tanja Vrijkotte, Mai J.M. Chinapaw

**Supervision:** Tanja Vrijkotte, Mai J.M. Chinapaw

**Writing–original draft:** Meredith L. Overman, Yolanda M. Sánchez Castro, Mai J.M. Chinapaw

**Writing–review & editing:** Meredith L. Overman, Tanja Vrijkotte, Yolanda M. Sánchez Castro, Margreet W. Harskamp-van Ginkel, Monica Hunsberger, Carry M. Renders, Stef P.J. Kremers, Mai J.M. Chinapaw
