# Supplementary material for: The cross-sectional association of parental psychosocial status with children’s Body Mass Index z-score and the mediating role of children’s energy balance behaviors - the ABCD Study
Source: PLoS One. 2024 Apr 29;19(4):e0302147. doi: 10.1371/journal.pone.0302147 (PMC11057752; doi:10.1371/journal.pone.0302147)
Supplement: Attachment — Submitted filename: Response to Reviewers.docx [file pone.0302147.s003.docx]

**Rebuttal Letter**

Subject: Re: PONE-D-23-29554 - Submission of Revised Manuscript

Dear Dr. Tilga and reviewers

We appreciate the opportunity to revise and resubmit the manuscript. Thank you for taking the time to review our manuscript and for your valuable questions and feedback regarding the manuscript. We appreciate your thorough review and we have carefully reviewed the comments the academic editor and reviewers provided and addressed each point thoroughly in our revised submission. This document addresses each point raised during the review process.

Kind regards, also on behalf of the co-authors

Meredith L. Overman, MSc
Department of Health Promotion, NUTRIM School of Nutrition and Translational Research in Metabolism, Maastricht University, Maastricht, Netherlands.

m.overman@maastrichtuniversity.nl

**Rebuttal**

| **Journal requirements** | **Response author** | **Changes made in manuscript** |
| --- | --- | --- |
| 1. Please ensure that your manuscript meets PLOS ONE's style requirements, including those for file naming. The PLOS ONE style templates can be found at  <https://journals.plos.org/plosone/s/file?id=wjVg/PLOSOne_formatting_sample_main_body.pdf> and  <https://journals.plos.org/plosone/s/file?id=ba62/PLOSOne_formatting_sample_title_authors_affiliations.pdf>  When submitting your revision, we need you to address these additional requirements. | We adjusted the following aspects in line with PLOS ONE’s style requirements:  We changed the filename for supporting information.  We adjusted the title page and the tables according to the PLOS ONE style templates. | File name supporting information: S1_Strobenut_Overman-ParentalPsychosocialStatusandBMI |
| 2. We note that you have indicated that data from this study are available upon request. PLOS only allows data to be available upon request if there are legal or ethical restrictions on sharing data publicly. For more information on unacceptable data access restrictions, please see <http://journals.plos.org/plosone/s/data-availability#loc-unacceptable-data-access-restrictions>.  In your revised cover letter, please address the following prompts:  a) If there are ethical or legal restrictions on sharing a de-identified data set, please explain them in detail (e.g., data contain potentially sensitive information, data are owned by a third-party organization, etc.) and who has imposed them (e.g., an ethics committee). Please also provide contact information for a data access committee, ethics committee, or other institutional body to which data requests may be sent.  b) If there are no restrictions, please upload the minimal anonymized data set necessary to replicate your study findings as either Supporting Information files or to a stable, public repository and provide us with the relevant URLs, DOIs, or accession numbers. For a list of acceptable repositories, please see <http://journals.plos.org/plosone/s/data-availability#loc-recommended-repositories>.  We will update your Data Availability statement on your behalf to reflect the information you provide. |  | Change made in cover letter and Plos One submission manager:  “Due to ethical considerations, the individual data from the ABCD study cannot be deposited in a public repository. This decision is guided by the need to protect the privacy and confidentiality of the study participants. The data contains potentially identifying or sensitive information about the participants, and releasing it publicly could breach their privacy rights. The ABCD data are owned by the Amsterdam University Medical Centers, location AMC in Amsterdam, The Netherlands. Any researcher can request the data by submitting a general publication proposal form to the ABCD Project Team as outlined at https://www.amc.nl/web/abcd-studie-2/abcd-studie/voor-onderzoekers-.htm under section use of data (Dutch: Gebruik van data), by email: abcd@amsterdamumc.nl. The ABCD Project Team will check proposals for compatibility with the general objectives, ethical approvals and informed consent forms of the ABCD study, that are in line with the guidelines laid down in the Declaration of Helsinki and all procedures involving research study participants approved by the Central Committee on Research Involving Human Subjects in The Netherlands; the medical ethics review committees of the Academic Medical Center, Amsterdam; the VU University Medical Center Amsterdam; and the Registration Committee of the Municipality of Amsterdam. There are no other restrictions to obtaining the data and all data requests will be processed in the same manner.” |

**Review Comments to the Author - Reviewer #1**

| **Review Comments to the Author - Reviewer #1** | **Response author** | **Changes made in manuscript**  **Concerning the line numbering, I refer to the line numbering in the Manuscript file, including track changes.**  **The changes that we made in the manuscript are in bold.** |
| --- | --- | --- |
| General comments The main issue with this manuscript is that statistical analyses do not match the study's aim, and the results do not support the authors' conclusion. I've described the reasons below. | Thank you for your comments. We aimed to answer all your questions as thoroughly as possible. We believe that your judgement regarding the mismatches of our analyses versus study aim and results versus conclusion are based on a misunderstanding. We kindly refer to our reply to these topics below. |  |
| Title 1. I prefer using "The cross-sectional association of ..." instead of " The association of ...". | Thank you for the suggestion. We adjusted the full title. | Change made in manuscript, line 1-2:  “The **cross-sectional** association of parental psychosocial status with children’s Body Mass Index **z-score** and the mediating role of children’s energy balance behaviors - the ABCD Study” |
| Abstract 2. Please indicate the results of mediation analyses on children’s diet, physical activity, and sleep. | Thank you for the suggestion.  We added the results of mediation analyses on children’s diet, physical activity, and sleep to the abstract. | Change made in manuscript, line 54-56: “**Children’s diet, physical activity, and sleep did not mediate the association between paternal psychosocial status and children’s BMI z-score**.” |
| Introduction 3. I disagree that educational level serves as a potential mediator. Parent educational levels are just a confounder, not a mediator. Current psychological stress never causes low socio-economic status. | We believe this comment is based on a misunderstanding. In our study we examined the potential moderating role of parental educational levels and NOT a mediating role. This is described in line 88-90. To prevent misunderstanding we added effect modifying in brackets. | Change made in manuscript, line 88-90: “As parents with higher levels of education tend to have children with healthier energy balanced-behaviors [21, 22] and lower BMI, we also examined the potential moderating **(i.e. effect modifying)** role of parental educational level.” |
| 4. In addition to the above comment, the authors appear to confuse mediation with effect modification. Please revise sentences in the introduction section distinguishing these concepts. | In addition to our previous answer, we examined the potential **moderating** role of parental educational level, not the mediating role. This is described in line 88-90. Moderation and effect modification are synonymous terms, referring to the same concept.  To prevent misunderstanding, we added “effect modifying” as a synonym for moderating in brackets.  See line 88-90 and line 231-233. | Change made in manuscript, line 88-90: “As parents with higher levels of education tend to have children with healthier energy balanced-behaviors [21,22] and lower BMI, we also examined the potential moderating **(i.e. effect modifying)** role of parental educational level.”  Change made in manuscript line 231-233: “In the final step, we conducted tests for moderated mediation, with the educational level of the respective parent serving as the **moderator (i.e. effect modifier)** in the association between parental psychosocial status and the potential mediators.” |
| Materials and methods 5. If the dietary quality score has been validated, please refer to a corresponding paper. If not, please describe the limitation of nonvalidating in the discussion section. | Thank you for raising this point. The diet quality score utilized in our study has not been validated. We have referred to a previous paper in the methodology section that utilizes the same diet quality score. Furthermore, we made some textual changes in the method section to clarify the origin of the Diet Quality Score.  We have added this limitation to the limitation section of our discussion in the manuscript in line 348-352. | Change made in manuscript, line 168-169: **“The ABCD study selected relevant items from existing questionnaires to assess children's food intake [28,29].”**  References:  28. Singh, A., et al., Design of the Dutch Obesity Intervention in Teenagers (NRG-DOiT). BMC Public Health, 2006. 6.  29. De Vries, S., et al., Kinderen in prioriteitswijken: lichamelijke (in) activiteit en overgewicht. 2005: Leiden: TNO.  Change made in manuscript, line 178-179: **“The calculation of the Diet Quality Score is in line with a study conducted as part of the broader ABCD study [30].”**  Reference:  30. Larsen, H., et al., Associations between parental and pre-adolescents' physical activity and diet quality: The role of parental child care involvement and child's sex. Eating Behaviors, 2023: p. 101775.  Change made in manuscript, line 355-358: **“However, this study utilized the Diet Quality Score to assess the consumption of fruits, vegetables, snacks, and sugar-sweetened beverages which has not been validated to date. However, the Diet Quality Score includes pivotal elements of children's dietary pattern that have been shown to be associated with future health [28,29]**  References:  28. Singh, A., et al., Design of the Dutch Obesity Intervention in Teenagers (NRG-DOiT). BMC Public Health, 2006. 6.  29. De Vries, S., et al., Kinderen in prioriteitswijken: lichamelijke (in) activiteit en overgewicht. 2005: Leiden: TNO. |
| 6. Please explain why children's age and sex were not included as covariates. | Thank you for your question. We calculated and analyzed BMI z-scores i.e. body mass index adjusted for age and sex of the child. So therefore children’s age and sex were not included as covariates again in the models as this would lead to inaccurate results.  We described this in line 148-149 in the method sections of the manuscript: “Each Child’s Body Mass Index was calculated (BMI z-score; calculated as weight (kg)/height (m2)) and converted into BMI z-scores, using age and sex adjusted reference tables [25].” |  |
| 7. In addition to comment 6, there can be other potential confounders. Referring to previous studies, please confirm covariates in this study are sufficient for analyses. | We carefully selected relevant confounders as described in the statistical analyses on page 10-11 and figures 2 and 3.  Covariates were organized in a DAG using DAGGity and a minimal adjustment set was identified: parental BMI; and DASS score of the other parent (see figures 2 and 3). |  |
| 8. Paternal and maternal psychosocial status should be adjusted simultaneously. This is because mothers whose partners have poorer psychosocial status tend to exhibit worse psychosocial status, the effect of paternal psychosocial status can include maternal ones, and vice versa. | We completely agree that adjusting paternal and maternal psychosocial status simultaneously is crucial and that is exactly what we did.  We mentioned this in the method section under statistical analyses in line 229-231: “Both models were adjusted for ethnicity (based on place of birth of the parents), parent BMI and the counter parent’s psychosocial status” |  |
| 9. The authors have a typo, misspelling "SPSS" as "SPS." | Thank you for noting this typo. We adjusted the typo from “SPS” to “SPSS”. | Change made in manuscript, line 220-221: “The final analysis includes complete **mother-father-child trios** and was performed using IBM **SPSS** package version 26.0 (**SPSS** Inc., Armonk, NY, USA).” |
| Results 10. Because the main exposures were not significantly associated with the outcome, the interpretation of the subsequent mediator analyses is questionable. If such interpretation is permissible, the authors must cite supporting literature. | We disagree with the reviewer on this point as methodological studies using empirical simulation have shown that the mediated effect may be statistically significant even when the total effect is not.  We kindly refer to:  O'Rourke HP, MacKinnon DP. Reasons for Testing Mediation in the Absence of an Intervention Effect: A Research Imperative in Prevention and Intervention Research. J Stud Alcohol Drugs. 2018 Mar;79(2):171-181. doi:10.15288/jsad.2018.79.171. PMID: 29553343; PMCID: PMC6019768.  We added this explanation and reference to the text. | Change made in manuscript, line 243-245: “**Furthermore, in this study, we consider mediation even if the association between the main exposure and outcomes is not statistically significant, as mediation analyses provide a more complete picture of associations by explaining working mechanisms** [35].”  Reference:  35. O'Rourke HP, MacKinnon DP. Reasons for Testing Mediation in the Absence of an Intervention Effect: A Research Imperative in Prevention and Intervention Research. J Stud Alcohol Drugs. 2018 Mar;79(2):171-181. doi:10.15288/jsad.2018.79.171. PMID: 29553343; PMCID: PMC6019768. |
| Discussion 11. The argument of this paper seems inconsistent with the results. Considering the lack of a significant association and the small beta from linear regression models, it would be accurate to conclude that there was no association between parental psychosocial status and children's Body Mass Index. | We are unsure which argument the reviewer is referring to here as we indeed report that we found no significant association between parental psychosocial status and children's BMI z-score.  See line 51-53 in the abstract:  “Maternal or paternal psychosocial status was not significantly associated with children’s BMI z-score (maternal β -0.0037; 95% CI: -0.008 to 0.0007, paternal β 0.0028; 95% CI: -0.007 to 0.002).”  See also line 267-269 in the beginning of the results section: “Neither maternal psychosocial status (β -0.0037; 95% CI: -0.008 to 0.0007) nor paternal psychosocial status (paternal β 0.0028; 95% CI: -0.007 to 0.002) were significantly associated with children’s BMI z-score” |  |

**Review Comments to the Author - Reviewer #2**

| **Review Comments to the Author - Reviewer #2** | **Response author** | **Changes made in manuscript**  **Concerning the line numbering, I refer to the line numbering in the Manuscript file, including track changes.** |
| --- | --- | --- |
| **General comments** |  |  |
| BMI is a different measurement to weight and using these terms interchangeably (for example in the short title) is both factually incorrect and misleading. | We completely agree with the reviewer and apologize for using the terms interchangeably.  We changed the short title. In addition we checked it throughout the manuscript and made adjustments accordingly | Change made in Plos One submission manager. Revised short title: “**Parental Psychosocial Status and Child Body Mass Index z-score.”**  Line 50-51: “The majority, 80%, of the parents were highly educated and born in the Netherlands, and 68% of the children had a **healthy BMI.”**  Line 61-62: “Keywords: parental psychosocial status, **BMI z-score**, energy-balance related behaviors, mediation analysis.”  Line 311-313: “Regarding the association between parental psychosocial status and children’s BMI, our findings are in line with Walton et al. [14] who found no significant association between parental stress and children’s **BMI z-score** either.”  Line 331-333: “Another explanation may be that children with a higher BMI z-score are more conscious of their dietary behaviors or parents may set more rules to prevent further **increases in the BMI z-score**.” |
| The use of very long sentences throughout makes this manuscript hard to read. | We acknowledge the importance of readability and have shortened sentences where relevant.  A native English speaker has checked our manuscript for readability and standard English, and made suggestions for improvement. |  |
| There are grammatical, nomenclature, and English language mistakes throughout the manuscript. | A native English speaker has checked our manuscript for standard English. |  |
| It was not mentioned if adjustment for multiple testing was performed. | Thank you for this remark.  We did not adjust for multiple testing but instead report effect sizes, confidence intervals, and p-values of all performed analyses to enable readers using their own judgment about the relevance of the observed associations. We have added this information to the analyses paragraph for transparency. | Change made in manuscript, line 240-242: “**We did not adjust for multiple testing but instead report effect sizes, confidence intervals, and p-values of all performed analyses to enable readers using their own judgment about the relevance of the observed associations.”** |
| **Concerns** |  |  |
| The BMI mathematical formula was devised based on the average Western European man’s physical characteristics and does not (1) distinguish between excess body fat, bone mass, or musculature, (2) interpret the distribution of fat (which is a predictor of negative health outcomes such as type 2 diabetes and heart disease), and (3) cannot distinguish between sex, age, or ethnicity. | Thank you for your elaborate remark with regard to the mathematical formula for BMI. We agree that BMI has its limitations and have added these limitations to the discussion section.   To clarify, in our study, see line 148-149 we used the BMI z-score i.e. BMI adjusted for age and sex of the child.  Line 148-149: “Each Child’s Body Mass Index was calculated (BMI z-score; calculated as weight (kg)/height (m2)) and converted into BMI z-scores, using age and sex adjusted reference tables [25]”.  Reference:  25. W.H.O. BMI z-scores for boys and girls from 5 to 19 years. 2007 [cited 2020 12-08-2020]; Available from: https://www.who.int/tools/growth-reference-data-for-5to19-years/indicators/bmi-for-age. | Change made in manuscript. Added sentences in discussion, page 18-19, line 343-347: “**BMI remains a valuable screening tool, especially at the population level, to identify individuals potentially at risk of being overweight or underweight. We used the BMI z-score i.e. BMI adjusted for age and sex of the child. However, it is important to acknowledge that BMI also has limitations. BMI lacks precision in distinguishing between muscle and fat mass and our reference values were not specific for ethnicity.”** |
| The authors highlight that this is a muti-ethnic study, however, the BMI formula’s inability to distinguish between ethnicities has not been addressed in the study.  Also age and sex adjusted reference tables were used to create the z-scores for the children, however, this was not done for the parents. | Thank you for your remark. As mentioned above we have added the limitations of BMI to the discussion section in the manuscript. See line 343-347.  We indeed did not create BMI z-scores for parents. Only for children, BMI is age and sex-specific because of variations in body composition due to their growth and development. We included the father's and mother's BMI as confounders in our study. | Change made in manuscript. Added sentences in discussion, page 18-19, line 343-347: “**BMI remains a valuable screening tool, especially at the population level, to identify individuals potentially at risk of being overweight or underweight. We used the BMI z-score i.e. BMI adjusted for age and sex of the child. However, it is important to acknowledge that BMI also has limitations. BMI lacks precision in distinguishing between muscle and fat mass and our reference values were not specific for ethnicity.”** |
| Furthermore, there is an ever-growing body of evidence showing that BMI should not be used as a standalone tool for categorising individuals as overweight or obese (or getting a full overview of an individual’s overall health). Rather, multiple measures should be considered, including: body adiposity index, waist circumference, and waist-to-hip index. | Thank you for raising this concern. We agree with your standpoint and therefore analyzed BMI z-scores as a continuous variable and we did not categorize individuals as normal weight or overweight. |  |
| The dietary quality score used in the study does not include the five standard food groups. Therefore, a comprehensive overview of the child’s diet is not used by the study. | Thank you for raising this point. We made some textual changes in the method section to clarify the origin of the Diet Quality Score. We have referred to a previous paper in the methodology section that utilizes the same diet calculation.  We have added this limitation to the limitation section of our discussion in the manuscript in line 353-356. | Change made in manuscript, line 168-169: “**The ABCD study selected relevant items from existing questionnaires to assess children's food intake [28,29]**  References:  28. Singh, A., et al., Design of the Dutch Obesity Intervention in Teenagers (NRG-DOiT). BMC Public Health, 2006. 6.  29. De Vries, S., et al., Kinderen in prioriteitswijken: lichamelijke (in) activiteit en overgewicht. 2005: Leiden: TNO.  Change made in manuscript, line 178-179: “**The calculation of the Diet Quality Score is in line with a previous study conducted as part of the broader ABCD study [30].”**  Reference:  30. Larsen, H., et al., Associations between parental and pre-adolescents' physical activity and diet quality: The role of parental child care involvement and child's sex. Eating Behaviors, 2023: p. 101775.  Change made in manuscript, line 353-356: “**However, this study utilized the Diet Quality Score to assess the consumption of fruits, vegetables, snacks, and sugar-sweetened beverages which has not been validated to date. However, the Diet Quality Score includes pivotal elements of children's dietary pattern that have been shown to be associated with future health [28,29]**  References:  28. Singh, A., et al., Design of the Dutch Obesity Intervention in Teenagers (NRG-DOiT). BMC Public Health, 2006. 6.  29. De Vries, S., et al., Kinderen in prioriteitswijken: lichamelijke (in) activiteit en overgewicht. 2005: Leiden: TNO. |
| The study only included sports participation as a measure of physical activity. This oversimplifies the possible physical activities the child could be performing on a regular basis, such as riding their bicycle or running. Furthermore, the parents’ diet or activity levels were not included, which is a massive limitation as shared family dynamic was not investigated. | Thank you for raising these valid points. We have added these limitations to the discussion section on page 19.  We agree that sports participations covers only a part of the total amount of physical activity. However, organized sport participation can be used as an indicator of overall moderate to vigorous physical activity.     - Wickel EE, Eisenmann JC. Contribution of youth sport to total daily physical activity among 6- to 12-yr-old boys. Med Sci Sports Exerc. 2007 Sep;39(9):1493-500. doi: 10.1249/mss.0b013e318093f56a. PMID: 17805079. - Machado-Rodrigues AM, Coelho e Silva MJ, Mota J, Santos RM, Cumming SP, Malina RM. Physical activity and energy expenditure in adolescent male sport participants and nonparticipants aged 13 to 16 years. J Phys Act Health. 2012 Jul;9(5):626-33. doi: 10.1123/jpah.9.5.626. Epub 2011 Jul 29. PMID: 21953445. - Hebert JJ, Møller NC, Andersen LB, Wedderkopp N. Organized Sport Participation Is Associated with Higher Levels of Overall Health-Related Physical Activity in Children (CHAMPS Study-DK). PLoS One. 2015 Aug 11;10(8):e0134621. doi: 10.1371/journal.pone.0134621. PMID: 26262678; PMCID: PMC4532417. - Marques, A., Ekelund, U., & Sardinha, L. B. (2016). Associations between organized sports participation and objectively measured physical activity, sedentary time and weight status in youth. *Journal of Science and Medicine in Sport*, *19*(2), 154-157. - Vella, S.A., Cliff, D.P., Okely, A.D. *et al.* Associations between sports participation, adiposity and obesity-related health behaviors in Australian adolescents. *Int J Behav Nutr Phys Act* **10**, 113 (2013). <https://doi.org/10.1186/1479-5868-10-113>.   We agree that understanding shared family dynamics related to energy balance behaviors is limited when you do not consider parents' dietary habits and physical activity. We reflected on this in the limitation section of the discussion. See line 350-352. | Change made in manuscript, line 347-350: “**Although sports participation is not a perfect measure of overall physical activity, organized sports participation is associated to overall moderate to vigorous physical activity as well as compliance with physical activity guidelines [41,42].”**  References:  41. Hebert JJ, Møller NC, Andersen LB, Wedderkopp N. Organized Sport Participation Is Associated with Higher Levels of Overall Health-Related Physical Activity in Children (CHAMPS Study-DK). PLoS One. 2015 Aug 11;10(8):e0134621. doi: 10.1371/journal.pone.0134621. PMID: 26262678; PMCID: PMC4532417.  42. Marques, A., Ekelund, U., & Sardinha, L. B. (2016). Associations between organized sports participation and objectively measured physical activity, sedentary time and weight status in youth. *Journal of Science and Medicine in Sport*, *19*(2), 154-157.  Change made in manuscript, line 350-352: “**In addition, we did not consider parents' dietary habits and physical activity. Hence, our understanding of shared family dynamics related to energy balance behaviors is limited.”** |
| Although the study has data on full mother-father-child trios the analyses were performed on parent-child dyads. The authors state that: “We included all parents who filled in the mother and father’s questionnaire, regardless of whether they were the biological parent or not. Our study does not include children with same sex parents or from single households.”. I have concerns about the authors choice to conduct analyses separately for each parent, rather than looking at the complete family. Furthermore, sensitivity analyses should have been performed for non-biological relationships to disentangle the direct and indirect familial effects. Finally, the exclusion of children from households with single parents and same-sex couples prevents the results to be generalisable to the general population. | We apologize for the confusion as we indeed analyzed the associations within the complete family by adjusting analyses of psychosocial status of the mother for the father and the other way around. We have clarified this by omitting dyad and using trios instead in the manuscript.  As no previous studies have examined the interplay between psychosocial status of both mother and father in relation to children’s energy balance behaviors and BMI z-score, we focused on mothers and fathers in this study. We acknowledge that exclusion of children from households with single parents and same-sex couples limits the generalizability of our study which we discussed in the limitations section, see line 360-361.  We added to the limitation of lacking information on biological parenthood in the limitations section, see line 361-362. | Change made in manuscript, line 135-139: “**Mother-father-child trios** with complete data for both parents and child were included. We included all parents who filled in the mother and father’s questionnaire, regardless of whether they were the biological parent or not. Our study does not include children with same sex parents or from single households. The final sample size included 1315 **mother-father-child trios**.”  Change made in manuscript, line 220-221: “The final analysis includes complete **mother-father-child trios** and was performed using IBM **SPSS** package version 26.0 (**SPSS** Inc., Armonk, NY, USA).”  Change made in manuscript, line 248-250: “In total, 1315 **mother-father-child trios** had complete data on children’s BMI z-score, dietary intake, sports participation, time in bed and screen time.”  Change made in manuscript, line  line 360-361: “Another limitation is that we **did not include data from households with single parents or** with same-sex parents.”  Change made in manuscript, line 361-362: “**Moreover, we lacked information on biological parenthood.”** |
| The authors state “Another major strength is the considerable sample size.”, however, the study included only 1,315 mother-father-child trios. Given this small sample size, I have considerable concerns about the statistical power the study has to identify a true effect of parental psychosocial status with children’s BMI (or the potential study mediators: children’s diet, sports participation, time in bed, and screen time). Furthermore, no replication analyses were performed, therefore, no evidence that the findings are reliable and applicable to the population as a whole were provided. | Thank you for your remark.  To address your concern about statistical power we added the a sentence to the analyses paragraph, see line 240-242.  Concerning the sample size, we believe that 1,315 mother-father-child trios is a considerable sample size and larger than previous studies on the association between the psychosocial status of mothers or fathers and the BMI of their children. In fact, as far as we know there is not one study with the inclusion of fathers, mothers and children. We addressed that also in the discussion of this manuscript in line 340-342.  With regard to the performance of replication analyses we call for such analyses in the recommendations section of the manuscript, see line 367-372. | Change made in manuscript, line 240-242: “**We did not adjust for multiple testing but instead report effect sizes, confidence intervals, and p values of all performed analyses to enable readers using their own judgment about the relevance of the observed associations”**  Change made in manuscript, see line 340-342: “**1,315 mother-father-child trios is a considerable sample size and larger than previous studies on the association between the psychosocial status of mothers or fathers and the BMI of their children** [7,9,10,12].”  References:  7. Marco, P.L., I.D. Valério, C.L.d.M. Zanatti, and H. Gonçalves, Systematic review: Symptoms of parental depression and anxiety and offspring overweight. Revista de Saúde Pública, 2020. 54.  9. Zarychta, K., et al., Parental Depression Predicts Child Body Mass via Parental Support Provision, Child Support Receipt, and Child Physical Activity: Findings From Parent/Caregiver–Child Dyads. Frontiers in psychology, 2020. 11: p. 161.  10. Jang, M., B. Owen, and D.R. Lauver, Different types of parental stress and childhood obesity: A systematic review of observational studies. Obesity Reviews, 2019. 20(12): p. 1740-1758.  12. O'Connor, S.G., et al., Associations of maternal stress with children's weight‐related behaviours: a systematic literature review. Obesity reviews, 2017. 18(5): p. 514-525.  Change made in manuscript, see line 367-372: “However, more research is needed to confirm and further explain the association between paternal psychosocial status and children’s BMI z-score through screen time. **Future research should be carried out** in more diverse**, at risk groups in order** to understand how parental psychosocial status might affect children’s BMI z-score through energy balance-related behaviors.” |
